# Tetralogy of Fallot Associated with Dysplastic Kidneys, Cloacal Anomalies, and Female Pseudohermaphroditism: A Systemic Anomaly of Septation?

**DOI:** 10.1155/2012/502919

**Published:** 2012-07-02

**Authors:** José Morales-Roselló, Teresa Escudero Serrano, Ana García Almela, Rafael Lázaro Santander

**Affiliations:** ^1^Servicio de Obstetricia y Ginecología, Hospital de la Plana, Villarreal, Spain; ^2^Servicio de Anatomía Patológica, Hospital de la Plana, Villarreal, Spain

## Abstract

A 20-week fetus was diagnosed with tetralogy of Fallot and multicystic kidneys. The postmortem study showed missing müllerian structures with small streak ovaries, external male genitalia, and an abnormal cloacal septation (imperforate anus with a sigmoid colon opening in the bladder). As the observed anomalies were related with septation, a mechanism related with the activation of specific growth factors, we discuss the possibility of a disorder in the function of the bone morphogenetic proteins as a common cause for the widespread anomalies found in this fetus.

## 1. Introduction

In the embryo heart, the conotruncal and atrioventricular cavities are divided by mean of endocardial cushions, forming the 4 chambers and the outflow tracts. Similarly, in the cloaca, a septation occurs when the mesoderm of the urorectal septum grows in an apical basal direction forming the bladder and the rectum. In the kidney, however, the opposite mechanism applies, as no septation is expected when the ureteric bud branches within the metanephric mesenchyme [1]. Septation is therefore an essential and widespread process in human development. Subsequently, any disruption due to excess or fault may cause malformation. Isolated anomalies of septation are frequently reported; however, when two or more septation disorders associate, a common etiology might exist. We present an example of this association in a 20-week fetus with cardiac, cloacal, and renal malformations and propose a plausible etiology for this sequence of malformations. 

## 2. Case Presentation

**Figure 1:**
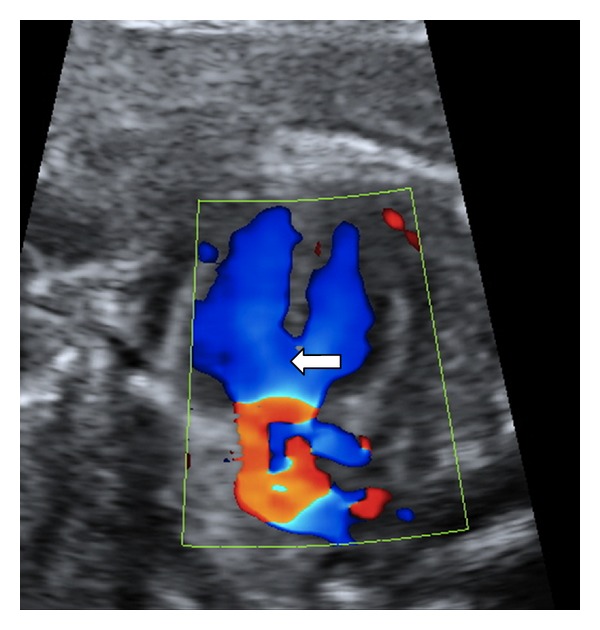
Color Doppler ultrasound showing the “Y sign”. Blood flow passes from the right and left ventricles, through the VSD (arrow), into an overriding enlarged aorta. Turbulent flow is shown as reddish areas around the aortic valve.

**Figure 2:**
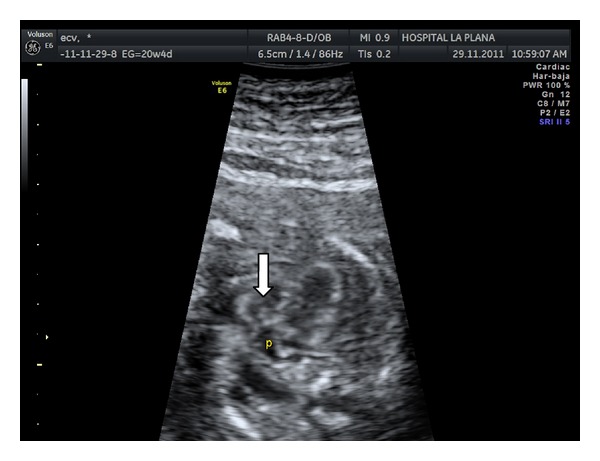
Small stenotic pulmonary vein (p) is seen connecting with the right ventricle (arrow).

**Figure 3:**
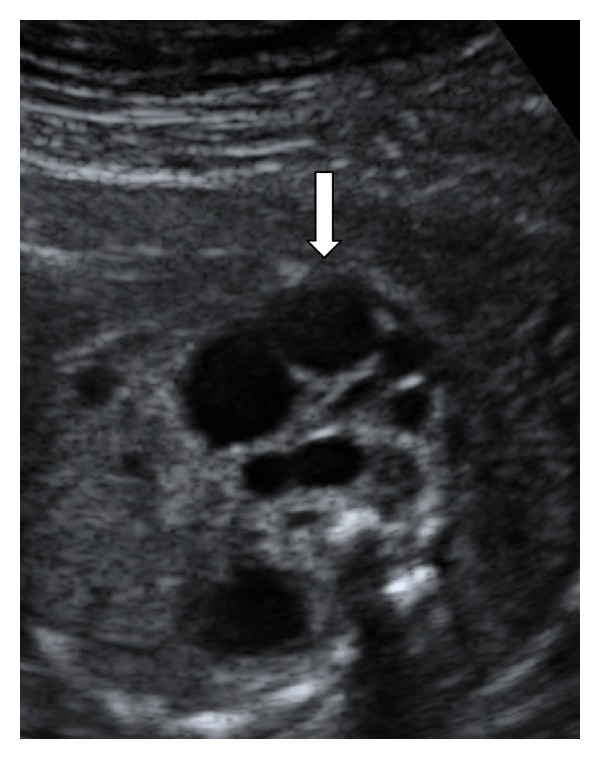
Transabdominal ultrasound showing a dysplastic (multicystic) kidney (arrow).

**Figure 4:**
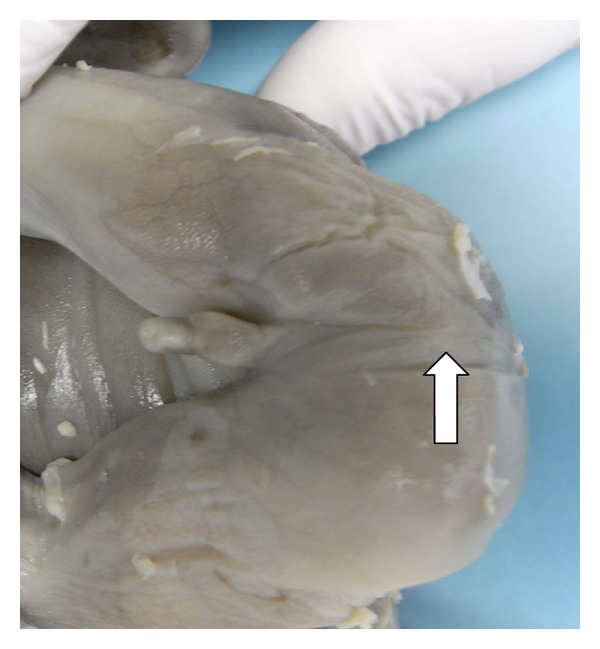
This female fetus showed masculine external genitalia with an imperforate anus (arrow).

**Figure 5:**
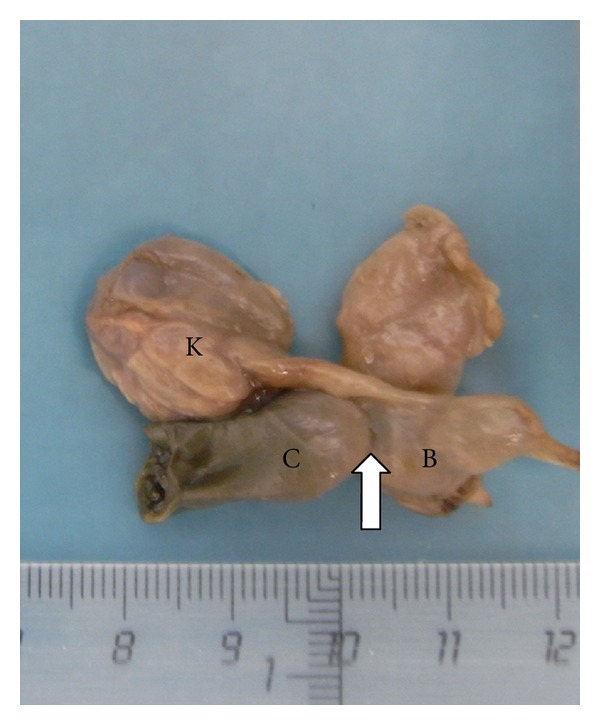
Cloacal septation anomaly. The sigmoid colon (C) ends in the bladder (B) (arrow). The ureters are short and connect with two dysplastic kidneys (K).

A 23-year-old woman in her first pregnancy with an ordinary follow-up and no previous medical history attended our ultrasound unit for a 20-week scan. The sonographic findings (levorotation of the cardiac axis, pericardial effusion, large overriding aorta, perimembranous ventricular septal defect, and narrow pulmonary artery) (Figures 1 and 2) diagnosed tetralogy of Fallot. The right atrium was slightly enlarged, and the fetus presented dysplastic (multicystic) kidneys with oligohydramnios (Figure 3). Due to the dreadful prognosis, the parents decided to terminate the pregnancy. The postmortem study confirmed the sonographic findings; however, it showed a right-sided aortic arch and an abnormal cloacal septation (imperforate anus with a sigmoid colon opening in the posterior side of the bladder) (Figures 4 and 5), which was not seen prenatally. Although the FISH showed only two copies of the X chromosome probe, with absence of a SRY marker, an external male genitalia with hypospadias was present (Figure 4). Internally, the uterus and fallopian tubes were missing and only two small streak ovaries with oogonia at both sides of the bladder were seen. Genetic test was also performed to rule out microdeletions in the chromosome 22 and mutations in the genes causing 21-hydroxylase-deficiency. All with negative results. 

## 3. Discussion

We were not able to find a common etiology for the association of malformations seen in this fetus; however, the observed anomalies seemed to be related with septation, a mechanism recently related with the progressive activation of specific growth factors. Although several molecules have been involved in this process, the bone morphogenetic proteins (BMPs), growth factors belonging to the ß-TGF superfamily, have become increasingly important. Concerning our case, they drew our attention as (1) in the heart, BMPs- and BMP-type II receptors are needed for septation of the outflow tract, and BMPs malfunction causes conotruncal anomalies [2], (2) in the embryonic cloaca, loss of BMP7 has been involved in the arrest of cloacal septation and genital defects [3], and (3) in the kidney, where branching of the ureteric bud depends on its inductive interactions with the metanephric mesenchyme, BMP-2,3,4,5, and 7 are expressed behaving as autocrine and paracrine molecules in the process of conducting renal development [4, 5]. 

BMPs and many other growth factors initiate, conduct, and finalize septation processes in the human body. It seems therefore plausible that any disturbance of the molecules in charge of these systemic processes may cause widespread anomalies of septation (heart, cloaca and kidneys). 

Also, we were unable to find a known etiology for the existence of a female pseudohermaphroditism as there was no history of hormonal intake, and the genetic tests for the SRY translocation and 21 hydroxylase deficiency were negative. However, anomalies of the cloaca in association with female pseudohermaphroditism have been previously reported [6–8], sometimes included in the urorectal septum malformation sequence [9]. For these associations, the BMPs may also have a role, as BMP7 has been involved in the formation of the urethra and genital tubercle [10].
